# Repeated gonadotropin stimulation modulates the expression of specific proteins in mouse oviduct

**DOI:** 10.5935/1518-0557.20250050

**Published:** 2025

**Authors:** Gianna Rossi, Sabrina Colafarina, Osvaldo Zarivi, Anna Maria Giuseppina Poma, Sandra Cecconi

**Affiliations:** 1 Department of Life, Health and Environmental Sciences, University of L’Aquila, 67100 L’Aquila, Italy

**Keywords:** gonadotropin, mouse, oviduct, oxidative stress, steroid receptors, p-53

## Abstract

**Objective:**

Superovulation protocol modifies the oviductal site-specific expression of some proteins regulating cell cycle and oxidative stress response.

**Methods:**

Swiss CD1 female mice (n=24) were sorted into 2 groups: one was used as control (Ctr, n=10), the other consisted of mice undergoing 8 Rounds (8R) of repeated gonadotropin stimulation (n=14). After their removal, oviducts were cut into two portions: one including Infundibulum and Ampulla (If-Am), and the rest including Isthmus (Is). Both portions were separately used to assess the expression levels of oviductal proteins regulating cell cycle and oxidative stress response. Statistical analysis employed t-test with significance at *p*<0.05.

**Results:**

In Ctr mice, superoxide dismutases 1 and 2 were significantly more expressed in the If-Am, while phospho-p53, glutathione peroxidase 1 and estrogen receptor beta mainly in the Is. Conversely, catalase, cleaved-caspase 3, estrogen alpha and progesterone receptors were similarly distributed across the oviduct. After 8R, glutathione peroxidase 1 and superoxide dismutase 1 increased in both segments, superoxide dismutase 2 and cleaved-caspase 3 increased mainly in If-Am, while catalase and phosphorylated p53 mainly in Is. Estrogen alpha/beta and progesterone receptors levels remained unchanged.

**Conclusions:**

Altogether, these results demonstrated that in the mouse oviduct many proteins were expressed in a site-specific manner and that repeated gonadotropin stimulation could modulate their expression levels. These data suggest that different localization of proteins between Infundibulum-Ampulla and Isthmus regions is fundamental for creating a suitable microenvironment for embryo development.

## INTRODUCTION

The mammalian oviduct, a tubular organ connecting ovary and uterus, makes a key contribution to fertilization and early steps of embryo development. The oviduct controls sperm survival, selection, and fertilizing capacity, influences embryo permanence and protects embryos from oxidative stress through the release of defensive proteins, such as heat shock proteins (HSP25 and 70) and antioxidant enzymes (Avilés *et al.,* 2015). The oviduct can be sorted in different segments characterized by specific morphological and molecular properties influenced by the proximity to uterus or ovary as well as by steroid hormone activity (Paik *et al.,* 2012). The existence of proteins differentially distributed across the oviduct indicates that oviductal cells sustain in a site-specific manner embryo development and its transition to uterus (Li & Winuthayanon, 2017).

The oviduct plays also a role in the onset of high-grade serous ovarian cancers (OC) (Sánchez-Prieto *et al.,* 2022; Cramer, 2023). The finding that these cancers express many oviductal genes (Kyo *et al.,* 2020) confirms the spreading of serous tubal intraepithelial carcinoma cells to ovarian surface (Meinhold-Heerlein *et al.,* 2016; Labidi-Galy *et al.,* 2017; Zhang *et al.,* 2019; Kyo *et al.,* 2020). Epidemiological data clearly show that OC incidence raises during the transition to menopause when women are exposed to high levels of gonadotropins, thus strongly supporting the role of incessant ovulation in the induction of this pathology (Hillier *et al.,* 2008). High gonadotropin levels can be transiently detected also in young women undergoing fertility treatments to increase the number of ovulated oocytes. The chance that these drugs could increase gynecological cancer risk has long been debated (Momenimovahed *et al.,* 2019) especially for OC (Farhud *et al.,* 2019; Rizzuto *et al.,* 2019). Luckly, almost all the published studies are reassuring because fertility drug treatments do not significantly increase the risk of invasive OC (Kroener *et al.,* 2017) also after repetitive cycles of hormonal stimulation (>4) (Brinton *et al.,* 2013). These drugs, however, can stimulate the onset of borderline OC that has a good prognosis when precociously diagnosed (Stewart *et al.,* 2013; Bjørnholt *et al.,* 2015).

By using a mouse model, we studied the effects of repeated rounds of administration of gonadotropins on oviductal protein expression, finding that these treatments modified the expression level of key cell cycle proteins (Di Luigi *et al.,* 2014; Di Nisio *et al.,* 2018) and of antioxidant enzymes (Di Nisio *et al.,* 2023). In particular, we found that after 8 rounds (8R) of hormonal stimulation ciliated cells of the ampullary region showed severe morphological changes (Antonouli *et al.,* 2020) and that the expression levels of specific proteins involved in cell cycle control p-AKT, cyclin D1, phosphorylated p53 (p-p53) and oxidative stress response catalase (CAT), glutathione peroxidase (GPx1), superoxide dismutases (SOD-1 and SOD-2), were significantly enhanced. All these data indicate that the 8R protocol can modify oviductal functions, yet they do not clarify if these changes occurred in the whole oviduct or only in specific segments.

The aim of this study was to investigate if 8R of gonadotropin stimulation could modulate the expression level of antioxidant enzymes (CAT, GPx1, SOD-1 and SOD-2), of estrogen alpha/beta (ERα, ERβ) and progesterone (PR) receptors, as well as of p-p53 and cleaved-caspase 3 (cCASP3) in two different segments of the mouse oviducts: one closer to ovaries and including infundibulum and ampulla (If-Am), and the other closer to uterus and including the isthmus (Is).

## MATERIALS AND METHODS

### Chemicals

All the chemicals used were purchased from the following sources: rabbit monoclonal Catalase (MAB-94599), SOD-1 (MAB-94600), SOD-2 (MAB-94601), Cleaved-caspase 3 p17 (D175) (AB-84283), mouse monoclonal GPx1 (MAB-94602) and ECL substrate (Pierce ECL-2001) from Immunological Sciences. Mouse monoclonal Estrogen receptor α (D12) (sc-8005), mouse monoclonal Estrogen receptor β (1531) (sc-53494) and rabbit polyclonal β-actin (sc-1616) were obtained from Santa Cruz Biotechnology (Santa Cruz, CA, USA). Mouse monoclonal Progesterone receptor (MA1-410) was obtained from Invitrogen (Waltham, MA, USA). Rabbit polyclonal phospho-p53(Ser15) (#9284), goat anti-rabbit IgG conjugated to horseradish peroxidase (HRP; #7074) and horse anti-mouse IgG conjugated to horseradish peroxidase (HRP; #7076), were obtained from Cell Signaling Technology (Beverly, MA, USA). All of the other reagents were purchased from Sigma-Aldrich (St. Louis, MO, USA), and were of the purest analytical grade.

### Collection of oviductal samples

Mus musculus Swiss CD1 adult female mice (2-3 months old; Harlan Italy, Udine, Italy) were housed in the animal facility under specific conditions (temperature:21±1°C; light:12 h light/day; food and water ad libitum). Female mice in early luteal phase of the estrous cycle, confirmed by vaginal examination (Cora *et al.,* 2015) were utilized in this study as Control (Ctr, n=10). Mice having 8 rounds of gonadotropin stimulation (8R; n=14) were obtained according to the protocol utilized in our previous studies (Di Luigi *et al.,* 2014; Di Nisio *et al.,* 2018). Oviducts taken from Ctr and stimulated groups were sorted into 2 portions: one closest to ovaries and including If-Am and one including the portion of Is. Samples were snap-frozen and stored at -80°C for further analysis. All the experiments were realized in conformity with national and international laws and policies (European Economic Community Council Directive 86/609, OJ 358, 1 12 December 1987; Italian Legislative Decree 116/92, Gazzetta Ufficiale della Repubblica Italiana n. 40, 18 February 1992; National Institutes of Health Guide for the Care and Use of Laboratory Animals, NIH publication no. 85-23, 1985). The project was approved by the Italian Ministry of Health and the Internal Committee of the University of L’Aquila (2017). All efforts were made to minimize suffering. The method of euthanasia consisted of an inhalant overdose of carbon dioxide (CO_2_, 10-30%), followed by cervical dislocation. All the biological material used in this study was archived material properly stored soon after collection.

### Western Blotting

For each sample, 20 µg of lysate were utilized for electrophoretic separation onto 10% or 12% gels under reducing conditions and transfer to nitrocellulose membranes (Hybond C Extra, Amersham). Membranes were incubated overnight at 4°C with specific primary antibodies in 5% w/v non-fat dry milk or in 5% w/v bovine serum albumin (BSA): anti-CAT (1:1000), anti-SOD-1 (1:1000), anti-SOD-2 (1:1000), anti-cCASP3 (1:500), anti-GPx1 (1:1000), anti-ERα (1:1000), anti-ERβ (1:1000), anti-phospho-p53 (1:1000) and anti-PR (1:1000). Secondary antibodies anti-rabbit IgG (HRP) and anti-mouse IgG (HRP) (diluted at 1:1000) were incubated with nitrocellulose membranes for 2 h at room temperature and peroxidase activity was revealed using a chemiluminescence reagent (ECL, Pierce). Membranes were examined by Alliance LD2-77WL imaging system (Uvitec, Cambridge, UK), and densitometric quantification was obtained with the public domain software NIH image v.1.62 after standardization using β-actin (1:2000) as a loading control.

### Statistical Analysis

All experiments were repeated at least 3 times, and data were expressed as the mean±SEM. Student’s t-test was used for comparison between the experimental groups. Results were considered statistically significant when *p<*0.05. All statistical analyses were performed using the statistical package SigmaPlot v.11.0 (Systat Software Inc., San Jose, CA, USA).

## RESULTS

**Figure 1:**
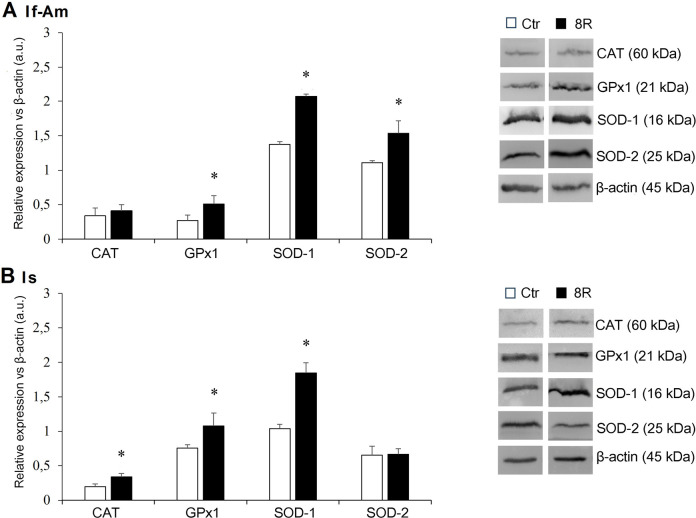
Antioxidant protein levels in A) If-Am and B) Is segments obtained from the oviducts of Ctr and 8R mice. Representative western blot images of CAT, GPx1, SOD-1 and 2 are reported in the figure. Values are expressed as arbitrary units (a.u.). Bar graph data represent the mean±SEM after normalization of each protein with respective β-actin used as a loading control for 4 independent determinations. *p<0.05 *vs*. Ctr.

The expression levels of oxidative stress proteins were determined in the segments of If-Am and Is, collected from either Ctr or 8R-stimulated mice. Data were reported in Figure 1 A and B, respectively. In Ctr mice, CAT was equally expressed across the oviduct, while SOD-1 and SOD-2 were more abundant in If-Am and GPx1 in Is (*p<*0.05). 8R mice displayed in both If-Am and Is more SOD-1 and GPx1 than Ctr mice, respectively (*p<*0.05). Conversely, SOD-2 level increased only in stimulated If-Am (Figure 1A; *p<*0.05 *vs.* Ctr), and CAT only in stimulated Is (Figure 1B; *p<*0.05 vs. Ctr).

**Figure 2:**
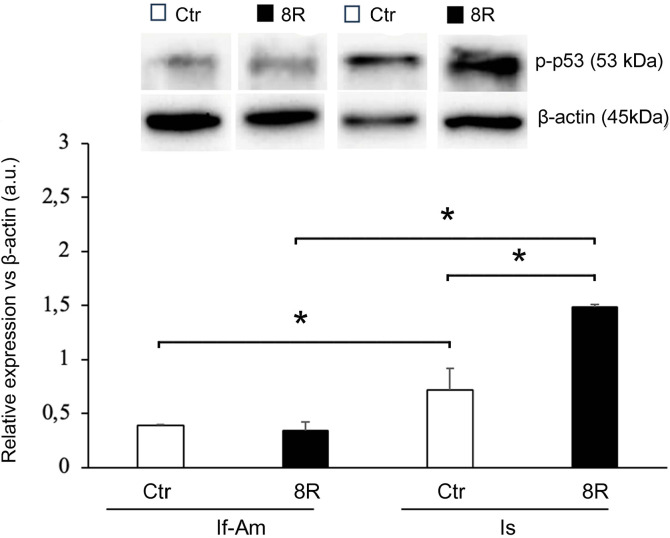
p-p53 level in the If-Am and Is segments retrieved from the oviducts of Ctr and 8R mice. Representative western blot images are reported in the figure. Values are expressed as arbitrary units (a.u.). Bar graph data represent the mean±SEM after normalization with β-actin used as a loading control for 3 independent determinations. *p<0.05.

As reported in Figure 2, in both Ctr and 8R mice p-p53 expression level was significantly higher in Is than If-Am (Ctr Is *vs*. Ctr If-Am, *p<*0.05; 8R Is *vs*. 8R If-Am, *p<*0.05). After 8R of gonadotropin stimulation, p-p53 expression was unchanged in If-Am (8R If-Am *vs*. Ctr If-Am: *p>*0.05), but significantly enhanced in Is (8R Is *vs*. Ctr Is, *p<*0.05).

**Figure 3:**
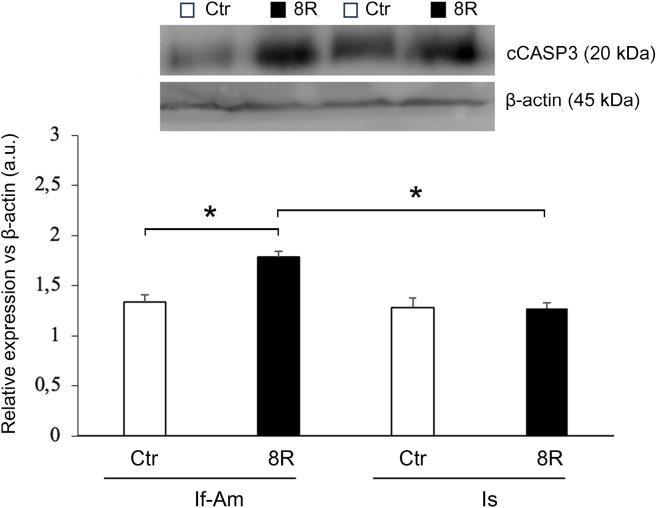
cCASP3 level in the If-Am and Is segments retrieved from the oviducts of Ctr and 8R mice. Representative western blot images are reported in the figure. Values are expressed as arbitrary units (a.u.). Bar graph data represent the mean±SEM after normalization with β-actin used as a loading control for 3 independent determinations. *p<0.05.

**Figure 4:**
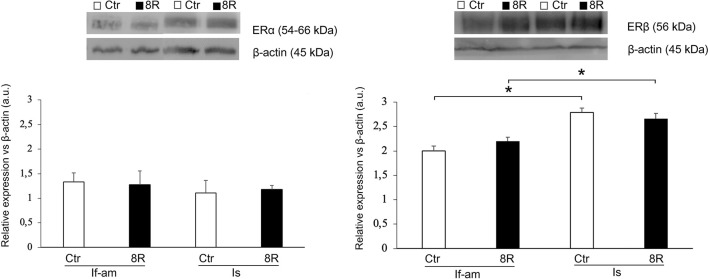
ERα and ERβ levels in the If-Am and Is segments retrieved from the oviducts of Ctr and 8R mice. Representative western blot images are reported in the figure. Values are expressed as arbitrary units (a.u.). Bar graph data represent the mean±SEM after normalization with β-actin used as a loading control for 3 independent determinations. *p<0.05.

The level of cCASP3 was comparable in both the segments of Ctr oviduct; conversely, after 8R a significant increase of this protein was recorded only in If-Am (Figure 3; Ctr *vs*. 8R: *p<*0.05; 8R If-Am *vs*. 8R Is, *p<*0.05). As for steroid receptors, similar ERα (Figure 4 A) and PR (not shown) contents were recorded in both oviductal segments, while ERβ was more abundantly expressed in Is than If-Am (Figure 4B; *p<*0.05). No significant modulation of receptor expression was observed upon gonadotropin stimulation.

## DISCUSSION

Literature data show that different regions of the oviduct express either different proteins or different levels of the same protein. For example, while WT1 and PAX2 are specific markers for the If-Am and Is segments respectively (Harwalkar *et al.,* 2021), the maintenance of a proper oviductal “anandamide tone”, needed for embryo transfer through the oviduct, requires the expression of a higher level of anandamide-hydrolase FAAH in the If-Am and of higher levels of anandamide-synthesizing NAPE-PLD in the Is (Schuel, 2006). Also, the shift from oxidative to glycolytic metabolism occurring from If-Am to Is segments is based on the differential expression of enzymes able to ensure the correct embryo metabolism and development in keeping with the maturation of embryonic mitochondria (Li & Winuthayanon, 2017). After ovulation, the If-Am segment, which is closer to ovary, is more exposed to a high oxidative stress. In mice, this stressful response is enhanced after repeated hormonal stimulation. In fact, mouse oviducts exposed to 8R of gonadotropins undergo changes in the expression level of several proteins, among which the antioxidant enzymes and p-p53 (Di Luigi *et al.,* 2014; Di Nisio *et al.,* 2018) as well as morphological alterations of ampullary ciliated cells and their mitochondria (Antonouli *et al.,* 2020).

In this study, we found that some selected proteins like p-p53, antioxidant enzymes (CAT, GPx1, SOD-1 and SOD-2), steroid hormone receptors (ERα, ERβ, PR) and cCASP3 are differentially expressed between If-Am and Is segments, and their levels are modulated by 8R of gonadotropin stimulation.

The fact that SOD-1 and 2 are more abundantly accumulated in If-Am is consistent with their closer proximity to the ovary. As above reported, these cells are exposed to a higher concentration of toxic substances accumulated in follicular fluid that are released during ovulation to alter oviductal surface (Chu & Shih, 2019). After 8R, the significant increase in SOD-1, SOD-2 and GPx1 expression recorded in If-Am confirms that a defensive response is elicited by repetitive ovulations. In our experiments, the increment of SOD-2 in If-Am occurs concomitantly with a slight, but not significant, decrease of p-p53. The existence of a reciprocal control on gene expression by which p53 is inversely correlated with SOD-2 expression has been hypothesized for the first time in transformed fibroblasts (Drane *et al*., 2001). This possibility is ruled out in our model by the observation that the sustained increment of p-p53 recorded in stimulated Is does not affect significantly SOD-2 levels. Therefore, the stimulation protocol adopted here is unlikely to lead to the initiation of cell transformation, because the increased expression of other antioxidant enzymes can control/reduce redox stress across the whole oviduct. Notably, as described in other cell systems (Handy & Loscalzo, 2022) we can hypothesize that p-p53 binding to *gpx1* promoter is responsible for GPx1 increase, which reinforces the defensive response against oxidative stress without triggering apoptosis (Hussain *et al.,* 2004).

As for cCASP3, we found that in Ctr mice it is equally expressed in both segments. Following 8R of stimulation, its increment in If-Am is too low to induce extensive apoptosis, because this process usually requires a sustained increase of cCASP3 expression (about 6 fold) (Lacoste *et al.,* 2013). Moreover, ultrastructural analysis showed the absence of terminal apoptotic features in the hyper-stimulated ampulla (Antonouli *et al.,* 2020). The fact that cCASP3 level is unchanged in Is confirms the major exposure of If-Am to ovulation-dependent damages. A low activation of cCASP3 could be involved in the regulation of other non-apoptotic functions, such as the shift from proliferative to differentiative status (Killinger *et al.,* 2021). We do not know if similar processes are activated in our experimental model, yet this shift might imply a role in the regeneration of epithelial cells after ovulation injury (Hsu *et al.,* 2019).

The crucial effects exerted by steroids on the regulation of the oviductal cell cycle are demonstrated by the findings that estrogen (E_2_) and progesterone (P_4_) regulate many functions of the oviduct such as inflammation, apoptosis, proliferation, contraction, and cytokine production (Almiñana *et al.,* 2018; Barton *et al.,* 2020). Regardless of stimulation, in our experiments ERα and PR are similarly expressed across the oviduct; conversely, ERβ expression is higher than that of the other receptors and more abundantly located in Is segment. These results confirm that repeated hormonal treatments do not significantly alter steroid-mediated signaling in oviductal cells. Indeed, the dysregulation of E_2_/ERα signaling is responsible for excessive inflammatory response and preimplantation embryo death in mice (McGlade *et al.,* 2022). The possibility that the higher expression of ERβ could exert a dominant negative effect on ERα-dependent cyclin D1 transcription (cannot be ruled out, because the increment of cyclin D1 content in the hyper-stimulated oviduct is well below its threshold level (Di Nisio *et al.,* 2018).

## CONCLUSION

In conclusion, our results contribute to a better understanding of the complexity of oviductal functions and properties, suggesting that a different localization of proteins between If-Am and Is regions is fundamental for creating a suitable microenvironment for embryo development. The modulation of these key proteins upon repeated cycles of gonadotropin stimulation reflects the capacity of different tracts of the oviduct to respond to region-specific stress conditions. A limit can be represented by the difficulty of confirming these results in the fallopian tubes of women undergoing different ranges of ovarian stimulations cycles. However, women genetically predisposed to OC onset should be carefully monitored because hormonal stimulations target also fallopian tubes that are a site of origin of this deadly cancer (George *et al.,* 2016).
